# Supplementary material for: Cyclin A2 degradation during the spindle assembly checkpoint requires multiple binding modes to the APC/C
Source: Nat Commun. 2019 Aug 27;10:3863. doi: 10.1038/s41467-019-11833-2 (PMC6712056; doi:10.1038/s41467-019-11833-2)
Supplement: Supplementary file 1 — Supplementary Information [file 41467_2019_11833_MOESM1_ESM.pdf]

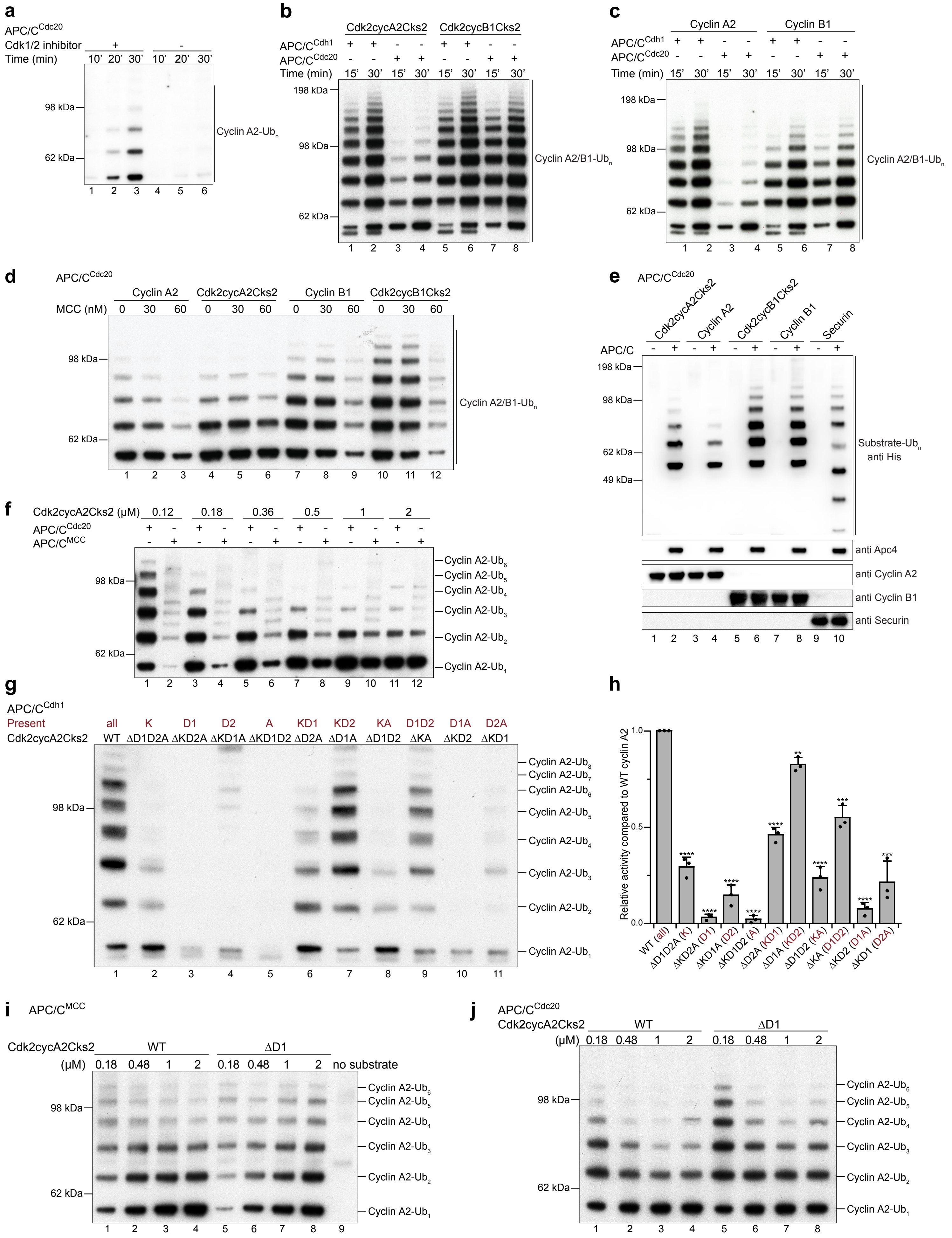

a
APC/C^Cdc20
Cdk1/2 inhibitor + -
Time (min) 10' 20' 30' 10' 20' 30'
98 kDa
62 kDa
Cyclin A2-Ub_n
b
Cdk2cycA2Cks2
Cdk2cycB1Cks2
APC/C^Cdh1
APC/C^Cdc20
Time (min) 15' 30' 15' 30' 15' 30' 15' 30'
198 kDa
98 kDa
62 kDa
Cyclin A2/B1-Ub_n
c
Cyclin A2
Cyclin B1
APC/C^Cdh1
APC/C^Cdc20
Time (min) 15' 30' 15' 30' 15' 30' 15' 30'
Cyclin A2/B1-Ub_n
d
APC/C^Cdc20
Cyclin A2
Cdk2cycA2Cks2
Cyclin B1
Cdk2cycB1Cks2
MCC (nM) 0 30 60 0 30 60 0 30 60 0 30 60
Cyclin A2/B1-Ub_n
e
APC/C^Cdc20
Cdk2cycA2Cks2
Cyclin A2
Cdk2cycB1Cks2
Cyclin B1
Securin
APC/C
198 kDa
98 kDa
62 kDa
49 kDa
Substrate-Ub_n anti His
anti Apc4
anti Cyclin A2
anti Cyclin B1
anti Securin
f
Cdk2cycA2Cks2 (μM) 0.12 0.18 0.36 0.5 1 2
APC/C^Cdc20
APC/C^MCC
Cyclin A2-Ub_6
Cyclin A2-Ub_5
Cyclin A2-Ub_4
Cyclin A2-Ub_3
Cyclin A2-Ub_2
Cyclin A2-Ub_1
g
APC/C^Cdh1
Present all K D1 D2 A KD1 KD2 KA D1D2 D1A D2A
Cdk2cycA2Cks2 WT ΔD1D2A ΔKD2A ΔKD1A ΔKD1D2 ΔD2A ΔD1A ΔD1D2 ΔKA ΔKD2 ΔKD1
Cyclin A2-Ub_8
Cyclin A2-Ub_7
Cyclin A2-Ub_6
Cyclin A2-Ub_5
Cyclin A2-Ub_4
Cyclin A2-Ub_3
Cyclin A2-Ub_2
Cyclin A2-Ub_1
h
Relative activity compared to WT cyclin A2
1.0
0.5
0.0
WT (all)
ΔD1D2A (K)
ΔKD2A (D1)
ΔKD1A (D2)
ΔKD1D2 (A)
ΔD2A (KD1)
ΔD1A (KD2)
ΔD1D2 (KA)
ΔKA (D1D2)
ΔKD2 (D1A)
ΔKD1 (D2A)
i
APC/C^MCC
Cdk2cycA2Cks2 WT ΔD1
(μM) 0.18 0.48 1 2 0.18 0.48 1 2 no substrate
Cyclin A2-Ub_6
Cyclin A2-Ub_5
Cyclin A2-Ub_4
Cyclin A2-Ub_3
Cyclin A2-Ub_2
Cyclin A2-Ub_1
j
APC/C^Cdc20
Cdk2cycA2Cks2 WT ΔD1
(μM) 0.18 0.48 1 2 0.18 0.48 1 2
Cyclin A2-Ub_6
Cyclin A2-Ub_5
Cyclin A2-Ub_4
Cyclin A2-Ub_3
Cyclin A2-Ub_2
Cyclin A2-Ub_1

**Supplementary Figure 1 | The resistance of cyclin A2 to MCC-imposed inhibition in vitro is concentration-dependent.** **a** Ubiquitination assay without addition of the Cdk1/2 inhibitor iii showed almost no APC/C activity. **b, c** Cyclin A2 and cyclin B1 in complex with Cdk2-Cks2 (**b**) or alone (**c**) are substrates of APC/C$^{Cdh1}$ and APC/C$^{Cdc20}$. **d** In contrast to cyclin B1, cyclin A2 was efficiently ubiquitinated in the presence of the MCC, but only when it was in complex with Cdk2-Cks2. **e** Control assay shows that in the absence of the APC/C, no ubiquitination ladder could be detected. **f** The resistance of cyclin A2 to MCC-imposed inhibition is concentration dependent. In addition, cyclin A2 showed a higher level of ubiquitination by APC/CCdc20 at low concentration and acts as an inhibitor at high concentration. **g, h** The KEN box plays a dominant role in cyclin A2 ubiquitination by APC/C$^{Cdh1}$, augmented by the D2 box. The combination of the KEN box and D2 box is sufficient to restore cyclin A2 ubiquitination by APC/C$^{Cdh1}$ approaching the same level as wild-type cyclin A2 (compare lanes 1 and 7). The Western-blot was quantified to show the effect of individual mutations, with error bars indicating standard deviation. The APC/C activity towards cyclin A2 mutants is normalized to ubiquitination of wild-type cyclin A2 and significance is calculated using unpaired Student's t-test (indicated with stars, Supplementary Table 2). **i, j** A titration of wild-type cyclin A2 and the ∆D1 mutant revealed that wild-type cyclin A2 was more efficient at overcoming MCC-imposed inhibition at low concentration (0.18-0.48 µM), indicating a role of the D1 box in cyclin A2 ubiquitination by APC/C$^{MCC}$ (**i**). This result is unique to APC/C$^{MCC}$, as for APC/C$^{Cdc20}$ a higher level of ubiquitination was observed for the ∆D1 mutant throughout the titration (**j**). Ubiquitination reactions were analysed by Western blotting with an anti-His antibody to detect the His-tag of the ubiquitin-modified substrates. Control gels showing the unmodified substrate for representative reactions are shown in Supplementary Fig. 2. Source data are provided as a Source Data file.

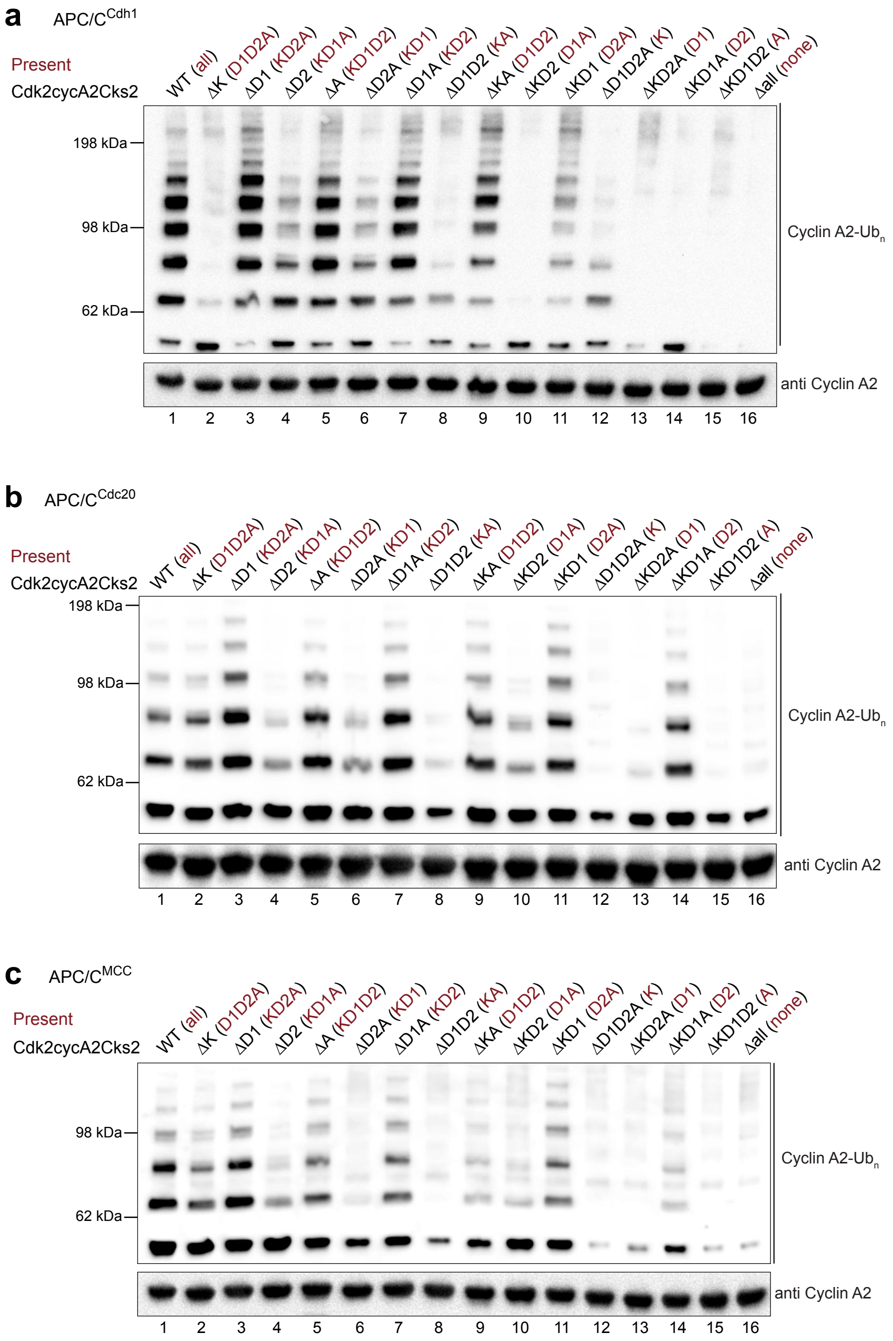


**Supplementary Figure 2 | Control assays of cyclin A2 mutants. a-c** Ubiquitination assays using APC/C$^{Cdh1}$ (**a**), APC/C$^{Cdc20}$ (**b**) and APC/C$^{MCC}$ (**c**) were performed for each cyclin A2 mutant. The modified substrates were detected by anti-His antibody against the His-tag on ubiquitin, while anti-cyclin A2 antibody detects the unmodified substrate. Source data are provided as a Source Data file.

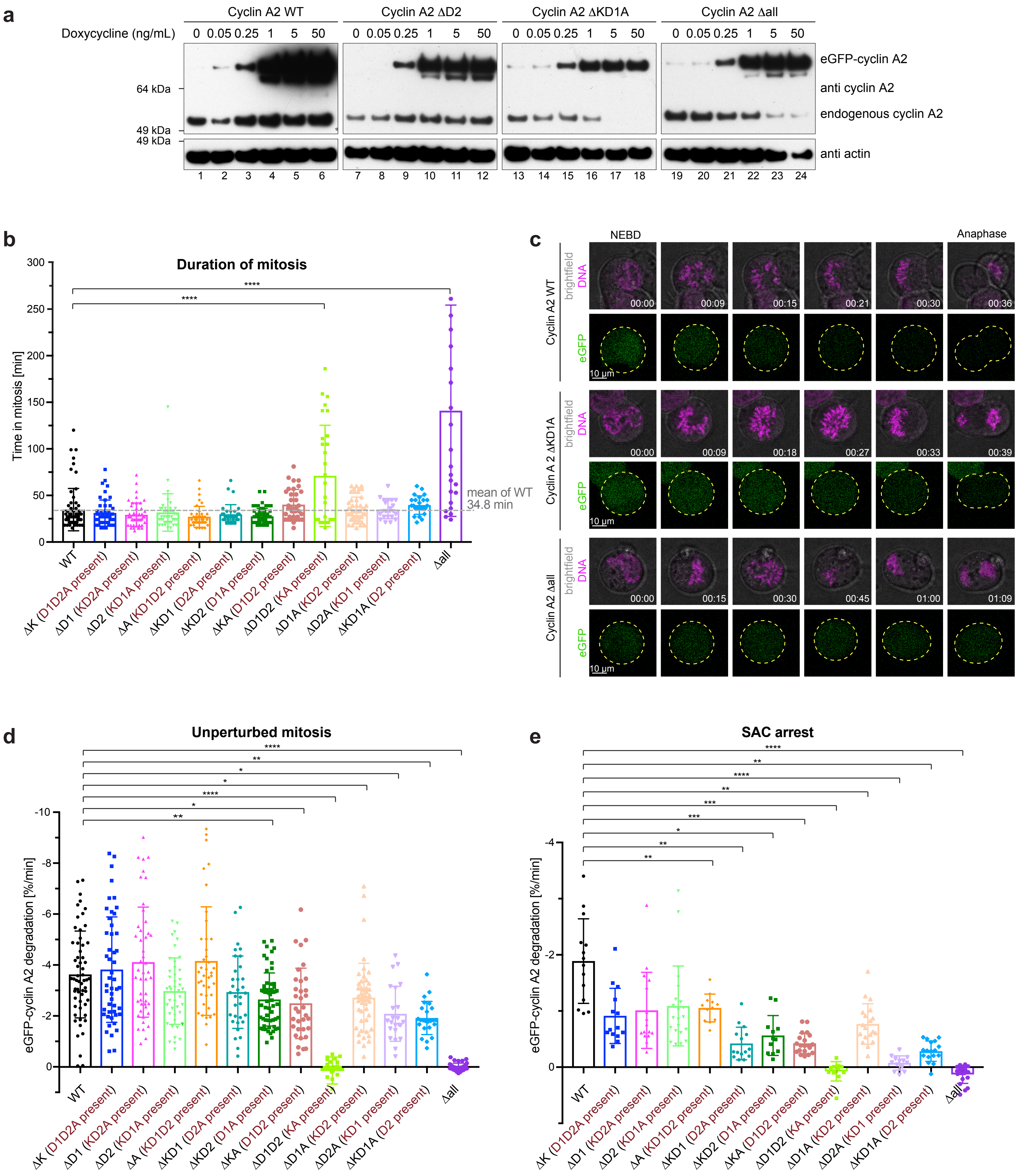
a
Cyclin A2 WT
Cyclin A2 ΔD2
Cyclin A2 ΔKD1A
Cyclin A2 Δall
Doxycycline (ng/mL) 0 0.05 0.25 1 5 50
64 kDa
49 kDa
49 kDa
eGFP-cyclin A2
anti cyclin A2
endogenous cyclin A2
anti actin
1 2 3 4 5 6 7 8 9 10 11 12 13 14 15 16 17 18 19 20 21 22 23 24
b
Duration of mitosis
Time in mitosis [min]
mean of WT 34.8 min
WT
ΔK (D1D2A present)
ΔD1 (KD2A present)
ΔD2 (KD1A present)
ΔA (KD1D2 present)
ΔKD1 (D2A present)
ΔKD2 (D1A present)
ΔKA (D1D2 present)
ΔD1D2 (KA present)
ΔD1A (KD2 present)
ΔD2A (KD1 present)
ΔKD1A (D2 present)
Δall
c
NEBD
Anaphase
Cyclin A2 WT
Cyclin A 2 ΔKD1A
Cyclin A2 Δall
brightfield DNA
eGFP
10 µm
d
Unperturbed mitosis
eGFP-cyclin A2 degradation [%/min]
e
SAC arrest
eGFP-cyclin A2 degradation [%/min]

**Supplementary Figure 3 | In vivo analysis of eGFP-cyclin A2 mutants. a** Immunoblot analysis of HEK293 FlpIn TRex cells expressing eGFP-cyclin A2 mutants after the addition of doxycycline. The expression was induced 48h before harvesting the cells and the concentrations of doxycycline are indicated. The membrane was blotted against cyclin A2, which detects both the endogenous protein as well as the eGFP-tagged constructs, and against actin as the loading control. **b** The duration of mitosis from NEBD to anaphase was measured for all cells analysed by live cell imaging. The expression of different eGFP-cyclin A2 mutants did not influence the time the cells spend in mitosis, except for ΔD1D2 and Δall. These two mutants are not targeted by the APC/C for degradation and therefore delay anaphase. Significance is calculated using a simple ANOVA test followed by Dunnett's multiple comparison test (Supplementary Table 2). **c** Exemplary still images from time courses between NEBD and anaphase of eGFP-cyclin A2 destruction in HEK cells of wild-type cyclin A2, ΔKD1A and Δall mutants. The chromosomes are coloured in magenta and cyclin A2 in green, with the outline of the cells marked with dashed yellow lines. Time is given as hh:mm. Scale bar 10 µm. Degradation of cyclin A2 was delayed to anaphase if only the D2 box was present (ΔKD1A) and it was completely inhibited if all degrons are mutated (Δall). The remaining degrons in cyclin A2 after the mutation are labelled in dark red. The complete time courses are shown in Supplementary Movies 3-5. **d, e** Degradation rate of the eGFP-cyclin A2 mutants was calculated during unperturbed mitosis (**d**) and SAC arrest (**e**). For each cell a linear regression over the linear part of the normalized degradation curve was performed. The slope of the regression line directly corresponds to the degradation of eGFP-cyclin A2 given as percent degradation per minute [%/min]. Significance is calculated by a simple ANOVA test followed by Dunnett's multiple comparison test (indicated with stars, Supplementary Table 2). Source data are provided as a Source Data file.

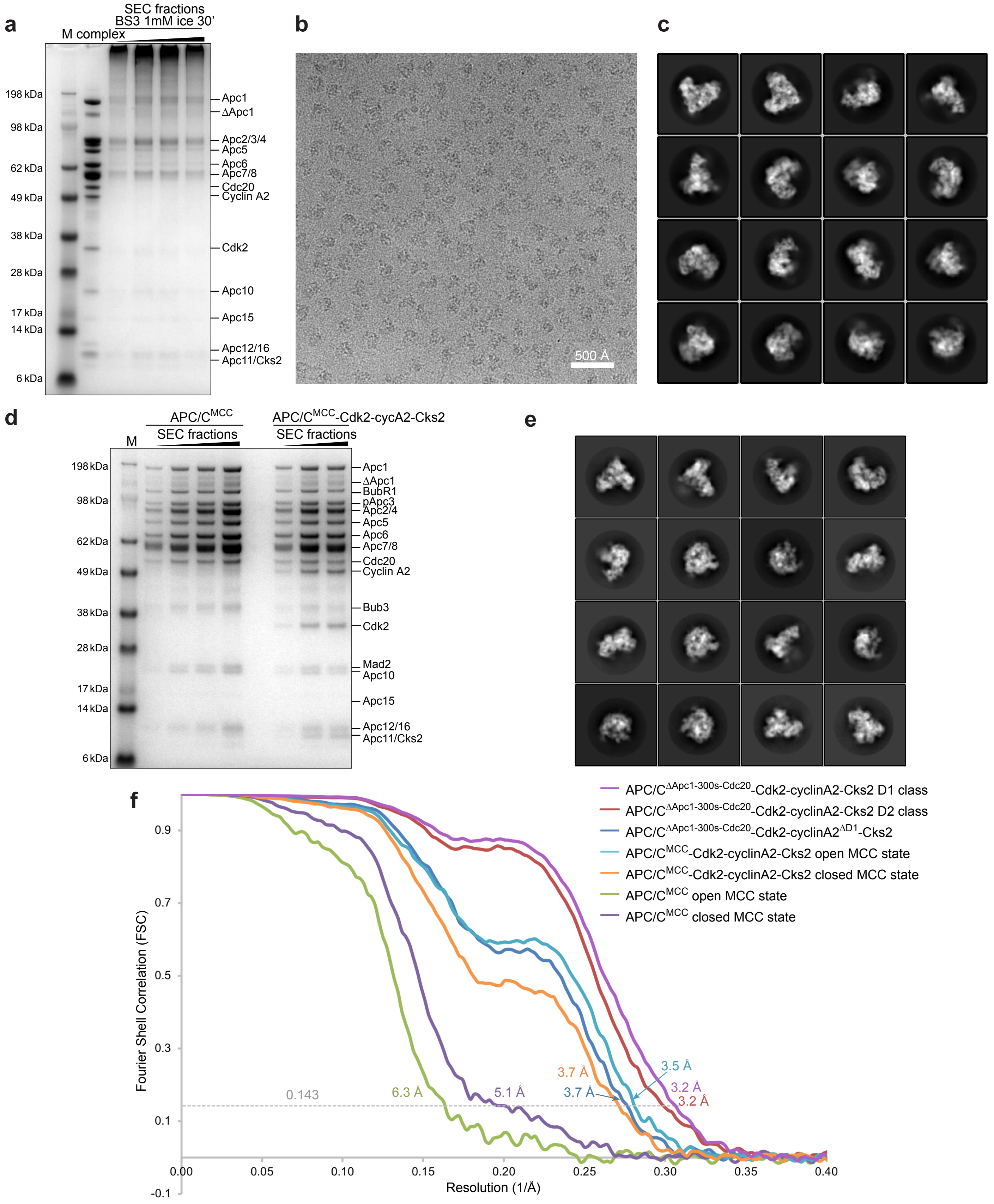


**Supplementary Figure 4 | Cryo-EM of APC/C$^{\Delta Apc1\text{-}300s\text{-}Cdc20}$-Cdk2-cyclinA2-Cks2 and APC/C$^{MCC}$-Cdk2-cyclinA2-Cks2.** **a** Purified APC/C$^{\Delta Apc1\text{-}300s\text{-}Cdc20}$-Cdk2-cyclinA2-Cks2 ternary complex on SDS-PAGE. Bands for individual APC/C subunits as well as Cdc20 and Cdk2-cyclinA2-Cks2 are indicated. A crosslinking condition using 1 mM BS3 on ice for 30 min was used. Crosslinked complex was purified by size-exclusion chromatography before grid preparation. **b** A typical cryo-EM micrograph of APC/C$^{\Delta Apc1\text{-}300s\text{-}Cdc20}$-Cdk2-cyclinA2-Cks2 collected with a FEI Titan Krios 300 kV microscope using the K2 detector in electron counting mode, representative of 8,264 micrographs collected. **c** Gallery of two-dimensional averages of APC/C$^{\Delta Apc1\text{-}300s\text{-}Cdc20}$-Cdk2-cyclinA2-Cks2 showing different views; representative of 100 two-dimensional averages. **d** Purified APC/C$^{MCC}$ and APC/C$^{MCC}$-Cdk2-cyclinA2-Cks2 complexes on SDS-PAGE. **e** Gallery of two-dimensional averages of APC/C$^{MCC}$-Cdk2-cyclinA2-Cks showing different views; representative of 100 two-dimensional averages. **f** Gold-standard Fourier Shell Correlation (FSC) curves of all APC/C reconstructions in this paper. Source data are provided as a Source Data file.

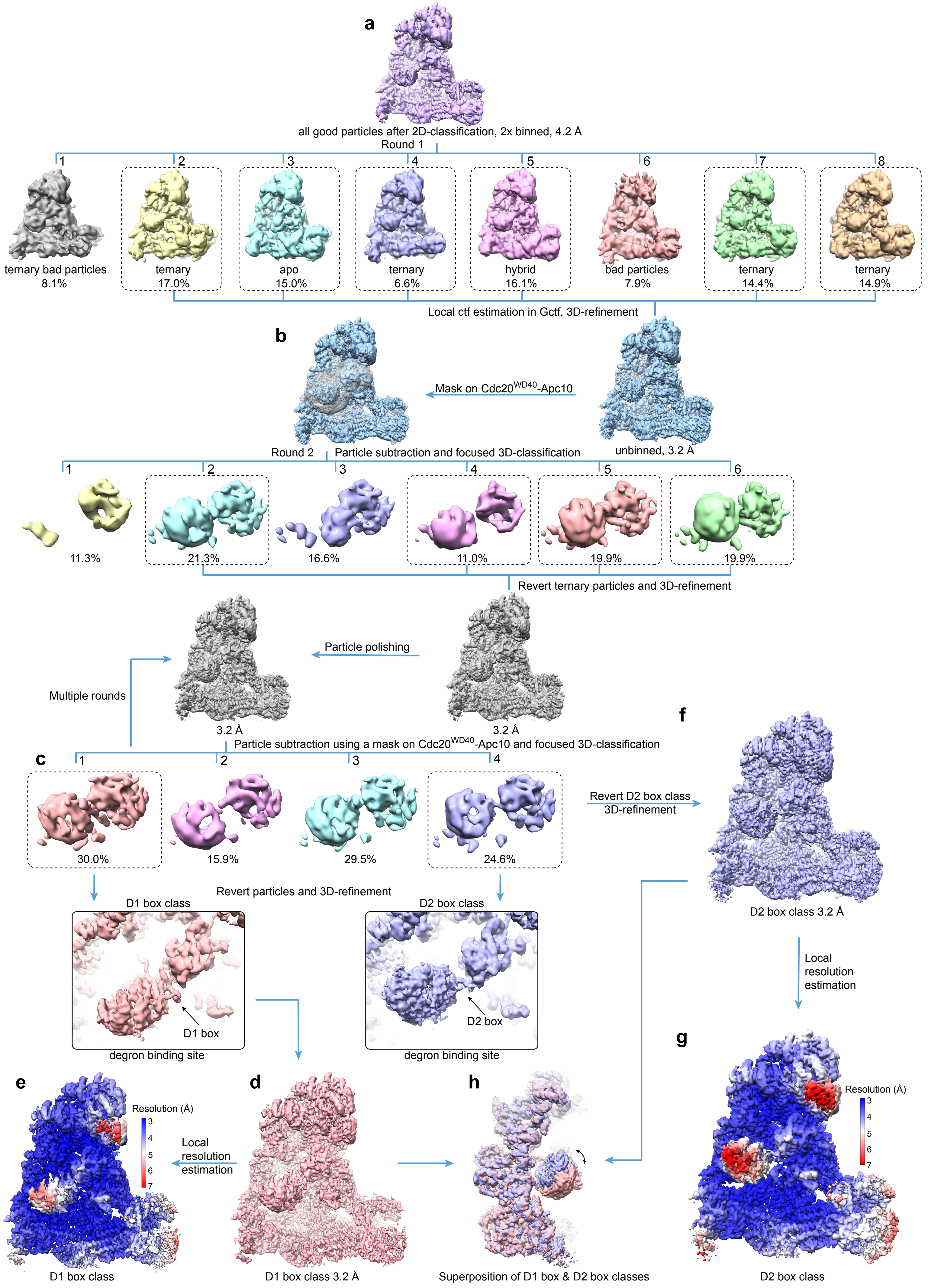

a
all good particles after 2D-classification, 2x binned, 4.2 Å
Round 1
1
2
3
4
5
6
7
8
ternary bad particles
8.1%
ternary
17.0%
apo
15.0%
ternary
6.6%
hybrid
16.1%
bad particles
7.9%
ternary
14.4%
ternary
14.9%
Local ctf estimation in Gctf, 3D-refinement
b
Mask on Cdc20^WD40-Apc10
unbinned, 3.2 Å
Round 2
Particle subtraction and focused 3D-classification
1
2
3
4
5
6
11.3%
21.3%
16.6%
11.0%
19.9%
19.9%
Revert ternary particles and 3D-refinement
Particle polishing
Multiple rounds
3.2 Å
3.2 Å
Particle subtraction using a mask on Cdc20^WD40-Apc10 and focused 3D-classification
c
1
2
3
4
30.0%
15.9%
29.5%
24.6%
Revert D2 box class
3D-refinement
f
D2 box class 3.2 Å
Revert particles and 3D-refinement
D1 box class
D2 box class
D1 box
D2 box
degron binding site
degron binding site
Local
resolution
estimation
g
Resolution (Å)
3
4
5
6
7
e
Resolution (Å)
3
4
5
6
7
Local
resolution
estimation
d
h
D1 box class
D1 box class 3.2 Å
Superposition of D1 box & D2 box classes
D2 box class

**Supplementary Figure 5 | Three-dimensional classification scheme of APC/C$^{\Delta Apc1\text{-}300s\text{-}Cdc20}$-Cdk2-cyclinA2-Cks2.** The initial particles after 2-dimensional classification were 3D-refined (**a**) and divided into eight classes by the 3-dimensional (3D) classification module using fine-angle search in RELION. Good particles including the apo (APC/C alone), ternary and hybrid (lacking Apc1$^{WD40}$) states were combined. Following local CTF correction in Gctf and 3D-refinement (**b**), a soft mask was applied on Cdc20$^{WD40}$-D box-Apc10 to perform particle subtraction and focused 3D-classification. Four out of the six classes contained densities for Cdc20$^{WD40}$ and were pooled for particle polishing to correct beam-induced particle motions. Multiple rounds of masking on Cdc20$^{WD40}$-D box-Apc10, focused 3D-classification and refinement were performed to separate different states of Cdc20. Two major classes containing Cdc20$^{WD40}$ and densities at the D-box binding site were obtained (**c**). The D1 box class contains a long-kinked loop density at the D-box binding site (**d**), whereas the D2 box class showed a canonical C-shaped density (**f**), with a large movement of Cdc20$^{WD40}$ relative to the D1 box class (**h**). Although the resolution of the APC/C in both classes extends to 3 Å, the resolution of the D-box binding site is limited to 4-5 Å (**e, g**). The class 3 in (**c**) (cyan) also contains the D1 box with strong densities for the ABBA motif and KEN box. However, there is a rotational movement of Cdc20$^{WD40}$ relative to the D1 box class (salmon), preventing merging the particles from the two classes.

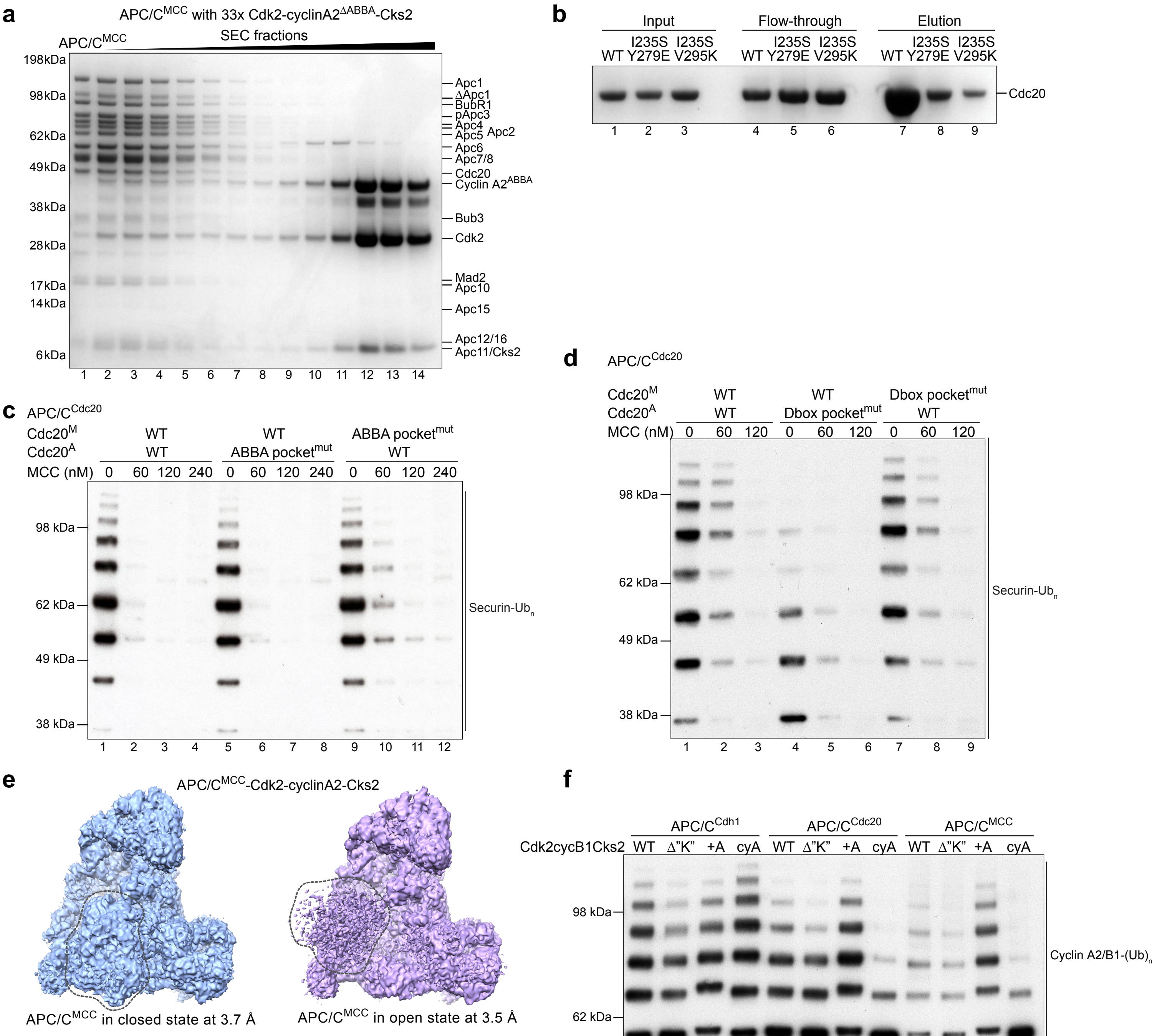


Ratio of the MCC in open-closed conformation

Particle numbers

| | Open | Closed | Ratio |
|---|---|---|---|
| APC/C$^{MCC}$ | 27 418 | 50 268 | 0.545:1 |
| APC/C$^{MCC}$-Cdk2-cyclinA2-Cks2 | 184 059 | 81 207 | 2.267:1 |

x4

**Supplementary Figure 6 | Control experiments and cryo-EM reconstruction of APC/C$^{MCC}$-Cdk2-cyclinA2-Cks2.** **a** The fractions of size exclusion chromatography of Cdk2-cyclinA2$^{\Delta ABBA}$-Cks2 with APC/C$^{MCC}$. Cdk2-cyclinA2$^{\Delta ABBA}$-Cks2 could bind to APC/C$^{MCC}$, but at a reduced level in comparison with wild-type cyclin A2. **b** A biotinylated peptide of cyclin A2 ABBA motif could efficiently pull-down wild-type Cdc20, whereas ABBA motif-binding pocket mutations with I235S and Y279E or I235S and V295K severely reduced association of the peptide (compare lanes 7 to 8-9). Mutations with I235S and V295K did not allow formation of the MCC. **c** In contrast to cyclin A2, mutation of the ABBA-motif binding pocket on Cdc20$^{M}$ reduced MCC-imposed inhibition of securin ubiquitination (lanes 9-12). **d** Disruption of the hydrophobic interactions within the D-box binding site (L176A) on Cdc20$^{M}$ did not influence the MCC-imposed inhibition on securin ubiquitination (lanes 7-9). **e** Comparison of the ratio between the open-closed APC/C$^{MCC}$ states in both cryo-EM reconstructions of APC/C$^{MCC}$ and APC/C$^{MCC}$-Cdk2-cyclinA2-Cks2. In the presence of Cdk2-cyclinA2-Cks2, a four-fold increase of the open state APC/C$^{MCC}$ was observed. The closed (cyan) and open (magenta) APC/C$^{MCC}$ states of the APC/C$^{MCC}$-Cdk2-cyclinA2-Cks2 reconstruction were determined to near-atomic resolution, with the densities corresponding to the MCC highlighted with dashed lines. **f** Mutation of the putative KEN box in cyclin B1 had no influences on its ubiquitination by APC/C$^{Cdc20}$ and APC/C$^{MCC}$, except for a small reduction for APC/C$^{Cdh1}$. Source data are provided as a Source Data file.

**Supplementary Table 1 | Statistics of cryo-EM reconstructions obtained in this paper.** Percentages in parenthesis indicate the proportion of good particles used for the final reconstruction.

| | APC/C$^{\Delta Apc1\text{-}300s}$-Cdc20-Cdk2-cyclinA2-Cks2 D1 box class (EMDB-4465) (PDB 6Q6G) | APC/C$^{\Delta Apc1\text{-}300s}$-Cdc20-Cdk2-cyclinA2-Cks2 D2 box class (EMDB-4466) (PDB 6Q6H) |
|---|---|---|
| **Data collection and processing** | | |
| Magnification | 130,000 | 130,000 |
| Voltage (kV) | 300 | 300 |
| Detector | Gatan K2 electron counting | Gatan K2 electron counting |
| Electron exposure (e–/Å$^2$) | ~28 | ~28 |
| Defocus range (μm) | 0.5-3.0 | 0.5-3.0 |
| Pixel size (Å) | 1.047 | 1.047 |
| Symmetry imposed | C1 | C1 |
| Initial particle images (no.) | 925,790 | 925,790 |
| Final particle images (no.) | 176,826 | 117,044 |
| Map resolution (Å) | 3.2 | 3.2 |
| FSC threshold | 0.143 | 0.143 |
| Map resolution range (Å) | 2.5-7 | 2.5-7 |
| **Refinement** | | |
| Initial model used (PDB code) | 4G04 | 4G04 |
| Map sharpening *B* factor (Å$^2$) | -30 | -30 |
| Model composition | | |
| Non-hydrogen atoms | 68,134 | 67,945 |
| Protein residues | 8,576 | 8,550 |
| *B* factors (Å$^2$) | | |
| Protein | 175.97 | 155.76 |
| R.m.s. deviations | | |
| Bond lengths (Å) | 0.007 | 0.007 |
| Bond angles (°) | 0.844 | 0.868 |
| Validation | | |
| MolProbity score | 1.63 | 1.64 |
| Clashscore | 6.14 | 5.94 |
| Poor rotamers (%) | 0.22 | 0.25 |
| Ramachandran plot | | |
| Favored (%) | 95.83 | 95.55 |
| Allowed (%) | 4.16 | 4.45 |
| Disallowed (%) | 0.01 | 0.00 |

| | APC/C$^{\Delta Apc1-300s}$-Cdc20-Cdk2-cyclinA2$^{\Delta D1}$-Cks2 (EMDB-4467) | APC/C$^{MCC}$-Cdc20-Cdk2-cyclinA2-Cks2 open MCC (EMDB-4463) | APC/C$^{MCC}$-Cdc20-Cdk2-cyclinA2-Cks2 closed MCC (EMDB-4464) |
|---|---|---|---|
| **Data collection and processing** | | | |
| Magnification | 105,000 | 93,000 | 93,000 |
| Voltage (kV) | 300 | 300 | 300 |
| Detector | Gatan K2 electron counting | FEI Falcon III integration | FEI Falcon III intergration |
| Electron exposure (e–/Å$^2$) | ~28 | ~28 | ~28 |
| Defocus range (μm) | 0.5-3.0 | 2.0-4.0 | 2.0-4.0 |
| Pixel size (Å) | 1.1 | 1.06 | 1.06 |
| Symmetry imposed | C1 | C1 | C1 |
| Initial particle images (no.) | 323,236 | 864,389 | 864,389 |
| Final particle images (no.) | 61,415 | 184,059 | 81,207 |
| Map resolution (Å) | 3.7 | 3.5 | 3.7 |
| FSC threshold | 0.143 | 0.143 | 0.143 |
| Map resolution range (Å) | 3-8 | 3-10 | 3-10 |

**Supplementary Table 2 | Statistical analysis of ubiquitination assays and fluorescence intensities.**
Details of the statistical analysis performed in this paper with p-values and significance.

Fig. 1c APC/C-Cdh1

| Unpaired t-test | Significant? | Summary | Adjusted P Value |
|---|---|---|---|
| dK (D1D2A present) vs. wt | Yes | **** | <0,0001 |
| dD1 (KD2A present) vs. wt | No | ns | 0,2663 |
| dD2 (KD1A present) vs. wt | Yes | ** | 0,0055 |
| dA (KD1D2 present) vs. wt | No | ns | 0,3233 |
| dAll vs. wt | Yes | **** | <0,0001 |

Fig. 1e APC/C-Cdc20

| Unpaired t-test | Significant? | Summary | Adjusted P Value |
|---|---|---|---|
| dK (D1D2A present) vs. wt | No | ns | 0,4226 |
| dD1 (KD2A present) vs. wt | No | ns | 0,1317 |
| dD2 (KD1A present) vs. wt | Yes | * | 0,039 |
| dA (KD1D2 present) vs. wt | Yes | ** | 0,0022 |
| dAll vs. wt | Yes | **** | <0,0001 |

Fig. 1g APC/C-MCC

| Unpaired t-test | Significant? | Summary | Adjusted P Value |
|---|---|---|---|
| dK (D1D2A present) vs. wt | Yes | * | 0,0347 |
| dD1 (KD2A present) vs. wt | No | ns | 0,4323 |
| dD2 (KD1A present) vs. wt | Yes | * | 0,0154 |
| dA (KD1D2 present) vs. wt | No | ns | 0,0807 |
| dAll vs. wt | Yes | **** | <0,0001 |

Fig. 3c APC/C-Cdc20

| Unpaired t-test | Significant? | Summary | Adjusted P Value |
|---|---|---|---|
| dKD1A (D2 present) vs. wt | No | ns | 0,058 |
| dKD1D2mutA vs. wt | Yes | ** | 0,0021 |
| dAll vs. wt | Yes | **** | <0,0001 |

Fig. 5b APC/C-Cdc20

| Unpaired t-test | Significant? | Summary | Adjusted P Value |
|---|---|---|---|
| dD1D2A (K present) vs. wt | Yes | **** | <0,0001 |
| dKD2A (D1 present) vs. wt | Yes | **** | <0,0001 |
| dKD1A (D2 present) vs. wt | Yes | * | 0,0113 |
| dKD1D2 (A present) vs. wt | Yes | **** | <0,0001 |
| dD2A (KD1 present) vs. wt | Yes | *** | 0,0002 |
| dD1A (KD2 present) vs. wt | Yes | **** | <0,0001 |
| dD1D2 (KA present) vs. wt | Yes | **** | <0,0001 |
| dKA (D1D2 present) vs. wt | Yes | ** | 0,0098 |
| dKD2 (D1A present) vs. wt | Yes | * | 0,0402 |
| dKD1 (D2A present) vs. wt | No | ns | 0,1342 |

Fig. 5d APC/C-MCC

| Unpaired t-test | Significant? | Summary | Adjusted P Value |
|---|---|---|---|
| dD1D2A (K present) vs. wt | Yes | **** | <0,0001 |
| dKD2A (D1 present) vs. wt | Yes | **** | <0,0001 |
| dKD1A (D2 present) vs. wt | Yes | ** | 0,0017 |
| dKD1D2 (A present) vs. wt | Yes | **** | <0,0001 |
| dD2A (KD1 present) vs. wt | Yes | *** | 0,0003 |

| | | | |
|---|---|---|---|
| dD1A (KD2 present) vs. wt | Yes | ** | 0,0053 |
| dD1D2 (KA present) vs. wt | Yes | **** | <0,0001 |
| dKA (D1D2 present) vs. wt | Yes | ** | 0,0026 |
| dKD2 (D1A present) vs. wt | Yes | * | 0,014 |
| dKD1 (D2A present) vs. wt | No | ns | 0,1182 |

Fig. 6f ABBA pocket mutant

| Unpaired t-test | Significant? | Summary | Adjusted P Value |
|---|---|---|---|
| wt (60 nM vs. 0 nM MCC) | No | ns | 0,2069 |
| wt (120 nM vs. 0 nM MCC) | No | ns | 0,0577 |
| Cdc20-A (60 nM vs. 0 nM MCC) | No | ns | 0,1321 |
| Cdc20-A (120 nM vs. 0 nM MCC) | No | ns | 0,1406 |
| Cdc20-M (60 nM vs. 0 nM MCC) | Yes | ** | 0,0086 |
| Cdc20-M (120 nM vs. 0 nM MCC) | Yes | * | 0,0337 |

Fig. 6h Dbox pocket mutant

| Unpaired t-test | Significant? | Summary | Adjusted P Value |
|---|---|---|---|
| wt (60 nM vs. 0 nM MCC) | No | ns | 0,4226 |
| wt (120 nM vs. 0 nM MCC) | No | ns | 0,0572 |
| Cdc20-A (60 nM vs. 0 nM MCC) | No | ns | 0,9003 |
| Cdc20-A (120 nM vs. 0 nM MCC) | No | ns | 0,2822 |
| Cdc20-M (60 nM vs. 0 nM MCC) | No | ns | 0,2047 |
| Cdc20-M (120 nM vs. 0 nM MCC) | Yes | * | 0,016 |

Fig. 7d cyclin B1 mutants

| Unpaired t-test | Significant? | Summary | Adjusted P Value |
|---|---|---|---|
| wt (60 nM vs. 0 nM MCC) | Yes | *** | 0,0007 |
| +K (60 nM vs. 0 nM MCC) | Yes | **** | <0,0001 |
| +Klinker (60 nM vs. 0 nM MCC) | Yes | ** | 0,0034 |
| +A (60 nM vs. 0 nM MCC) | Yes | * | 0,0458 |
| +KlinkerA (60 nM vs. 0 nM MCC) | Yes | ** | 0,0084 |
| Cdk2cycA2Cks2 (60 nM vs. 0 nM MCC) | No | ns | 0,4226 |

Supplementary Fig. 1h APC/C-Cdh1

| Unpaired t-test | Significant? | Summary | Adjusted P Value |
|---|---|---|---|
| dD1D2A (K present) vs. wt | Yes | **** | <0,0001 |
| dKD2A (D1 present) vs. wt | Yes | **** | <0,0001 |
| dKD1A (D2 present) vs. wt | Yes | **** | <0,0001 |
| dKD1D2 (A present) vs. wt | Yes | **** | <0,0001 |
| dD2A (KD1 present) vs. wt | Yes | **** | <0,0001 |
| dD1A (KD2 present) vs. wt | Yes | ** | 0,0012 |
| dD1D2 (KA present) vs. wt | Yes | **** | <0,0001 |
| dKA (D1D2 present) vs. wt | Yes | *** | 0,0002 |
| dKD2 (D1A present) vs. wt | Yes | **** | <0,0001 |
| dKD1 (D2A present) vs. wt | Yes | *** | 0,0002 |

| Supplementary Fig. 3b | Mitotic timing | | |
|---|---|---|---|
| Dunnett's multiple comparisons test | Significant? | Summary | Adjusted P Value |
| wt vs. dK (D1D2A present) | No | ns | 0,9991 |
| wt vs. dD1 (KD2A present) | No | ns | 0,9842 |
| wt vs. dD2 (KD1A present) | No | ns | 0,9994 |
| wt vs. dA (KD1D2 present) | No | ns | 0,8998 |
| wt vs. dKD1 (D2A present) | No | ns | 0,9958 |
| wt vs. dKD2 (D1A present) | No | ns | 0,8709 |

| | | | |
|---|---|---|---|
| wt vs. dKA (D1D2 present) | No | ns | 0,9929 |
| wt vs. dD1D2 (KA present) | Yes | **** | <0.0001 |
| wt vs. dD1A (KD2 present) | No | ns | >0.9999 |
| wt vs. dD2A (KD1 present) | No | ns | >0.9999 |
| wt vs. dKD1A (D2 present) | No | ns | 0,9991 |
| wt vs. dAll | Yes | **** | <0.0001 |

| Supplementary Fig. 3d | Degradation rate mitosis | | |
|---|---|---|---|
| Dunnett's multiple comparisons test | Significant? | Summary | Adjusted P Value |
| wt vs. dK (D1D2A present) | No | ns | 0,9994 |
| wt vs. dD1 (KD2A present) | No | ns | 0,7952 |
| wt vs. dD2 (KD1A present) | No | ns | 0,3594 |
| wt vs. dA (KD1D2 present) | No | ns | 0,7939 |
| wt vs. dKD1 (D2A present) | No | ns | 0,3398 |
| wt vs. dKD2 (D1A present) | Yes | ** | 0,0095 |
| wt vs. dKA (D1D2 present) | Yes | * | 0,0213 |
| wt vs. dD1D2 (KA present) | Yes | **** | <0.0001 |
| wt vs. dD1A (KD2 present) | Yes | * | 0,0224 |
| wt vs. dD2A (KD1 present) | Yes | * | 0,0288 |
| wt vs. dKD1A (D2 present) | Yes | ** | 0,0016 |
| wt vs. dAll | Yes | **** | <0.0001 |

| Supplementary Fig. 3e | Degradation rate SAC | | |
|---|---|---|---|
| Dunnett's multiple comparisons test | Significant? | Summary | Adjusted P Value |
| wt vs. dK (D1D2A present) | No | ns | 0,093 |
| wt vs. dD1 (KD2A present) | No | ns | 0,0887 |
| wt vs. dD2 (KD1A present) | No | ns | 0,0986 |
| wt vs. dA (KD1D2 present) | Yes | ** | 0,0081 |
| wt vs. dKD1 (D2A present) | Yes | ** | 0,0071 |
| wt vs. dKD2 (D1A present) | Yes | * | 0,048 |
| wt vs. dKA (D1D2 present) | Yes | *** | 0,0002 |
| wt vs. dD1D2 (KA present) | Yes | *** | 0,0001 |
| wt vs. dD1A (KD2 present) | Yes | ** | 0,0036 |
| wt vs. dD2A (KD1 present) | Yes | **** | <0.0001 |
| wt vs. dKD1A (D2 present) | Yes | ** | 0,0019 |
| wt vs. dAll | Yes | **** | <0.0001 |

ns not significant
