# Supplementary material for: Cyclin A2 degradation during the spindle assembly checkpoint requires multiple binding modes to the APC/C
Source: Nat Commun. 2019 Aug 27;10:3863. doi: 10.1038/s41467-019-11833-2 (PMC6712056; doi:10.1038/s41467-019-11833-2)
Supplement: Supplementary file 3 — Description of Additional Supplementary Files [file 41467_2019_11833_MOESM3_ESM.pdf]

Description of Additional Supplementary Files

File name: Supplementary Movie 1.
Description: Live cell imaging of wild-type cyclin A2 degradation under unperturbed mitosis for comparison with Supplementary Video 2. Full movie for the exemplary images shown in Figure 1j (top two rows). DNA is shown in magenta together with a bright field image of the cell and eGFP-Cyclin A2 wild-type in green. Time is given as hh:mm.

File name: Supplementary Movie 2.
Description: Live cell imaging of the cyclin $A2^{\Delta D2}$ mutant degradation under unperturbed mitosis. Full movie for the exemplary images shown in Figure 1j (bottom two rows). DNA is shown in magenta together with a bright field image of the cell and eGFP-Cyclin A2 ΔD2 in green. Time is given as hh:mm.

File name: Supplementary Movie 3.
Description: Live cell imaging of wild-type cyclin A2 degradation under unperturbed mitosis for comparison with Supplementary Videos 4, 5.
Full movie for the exemplary images shown in Supplementary Figure 3c (top two rows). DNA is shown in magenta together with a bright field image of the cell and eGFP-Cyclin A2 wild-type in green. Time is given as hh:mm.

File name: Supplementary Movie 4.
Description: Live cell imaging of the cyclin $A2^{\Delta KD1A}$ mutant degradation under unperturbed mitosis. Full movie for the exemplary images shown in Supplementary Figure 3c (middle two rows). DNA is shown in magenta together with a bright field image of the cell and eGFP-Cyclin A2 ΔKD1A in green. Time is given as hh:mm.

File name: Supplementary Movie 5.
Description: Live cell imaging of the cyclin $A2^{\Delta all}$ mutant degradation under unperturbed mitosis. Full movie for the exemplary images shown in Supplementary Figure 3c (bottom two rows). DNA is shown in magenta together with a bright field image of the cell and eGFP-Cyclin A2 Δall in green. Time is given as hh:mm.
